# Comparison of auditory cueing in toe tapping and gait in persons with Parkinson’s disease

**DOI:** 10.3389/fnhum.2023.1197247

**Published:** 2023-09-01

**Authors:** Elizabeth L. Stegemöller, Riley Berg, Alison Warnecke, Mollie Hammer

**Affiliations:** Department of Kinesiology, Iowa State University, Ames, IA, United States

**Keywords:** bradykinesia, gait, cueing, repetitive movement, Parkinson’s disease

## Abstract

**Introduction:**

Much research has examined the relationship between bradykinesia and gait impairment in persons with Parkinson’s disease (PD). Specifically, impairments in repetitive movements of the upper extremity have been associated with freezing of gait. Studies examining lower extremity repetitive movements are limited. Moreover, the use of external cueing has been a treatment strategy for both bradykinesia and gait, but information on how cues should be used is lacking. The purpose of this study was to compare the effects of auditory cueing on one side versus both sides for bilateral repetitive toe tapping and gait, and to determine if there was a relationship between toe tapping and gait. We hypothesize that there will be no difference between the cueing conditions, but that there will be a significant association between repetitive toe tapping performance and gait performance.

**Methods:**

Twenty-seven persons with PD completed a toe tapping task in which the more affected side was cued at 70 beats per minute (BPM), the less affected side was cued at 70 BPM, and both sides were cued at 140 BPM. The same cueing conditions were completed for the gait task. Inter movement interval and amplitude data was collected and analyzed for the toe tapping task. Stance time, swing time, step length, and step width were collected and analyzed for the gait task.

**Results:**

Results revealed a significant difference in movement performance between the single side cueing conditions and both sides cued condition for inter movement interval (toe tapping), stance time (gait), step length (gait), and step width (gait). Moreover, results revealed a significant association between inter movement interval and stance time and step length.

**Discussion:**

These results would suggest that cueing both sides is better than only one side and that there is a relationship between toe tapping and gait performance when both sides are cued in persons with PD. This study adds to the literature exploring possible shared mechanisms between bradykinesia and gait in persons with PD.

## 1. Introduction

Among the major clinical signs of Parkinson’s disease (PD), bradykinesia (a slowed ability to start and continue movements) is considered to have the greatest impact on functional disability (Rocchi et al., 2002; Diederich et al., 2003) and is the clinical parameter best correlated with disease severity and rate of progression of the disease (Agostino et al., 2003). Repetitive movements of both the upper and lower limbs are tasks used to assess bradykinesia in the clinic (4 out of 14 standardized motor tests of the United Parkinson’s Disease Rating Scale, UPDRS). Thus, more recent research has begun to explore how repetitive movement performance relates to functional movement tasks, such as walking, in persons with PD.

Much research has examined the relationship between repetitive finger tapping and freezing of gait (FOG) in persons with PD, suggesting that a common underlying mechanism is shared between freezing-like episodes in finger tapping and FOG. Vercruysse and colleagues have shown that small amplitude movements trigger both upper limb freezing and FOG, and that those with FOG showed greater dyscontrol of alternating bimanual repetitive finger movements compared to those without FOG (Vercruysse et al., 2012a,b). Moreover, freezing-like episodes in antiphasic finger tapping tend to be more pronounced among freezers than non-freezers (Barbe et al., 2014). However, research remains equivocal as to whether there is a statistical association between freezing-like episodes in finger tapping and FOG with some studies providing evidence that there is an association while others showing no association (Nieuwboer et al., 2009; Barbe et al., 2014). While there is promising evidence comparing finger tapping and gait/FOG, research comparing other repetitive movements, such as toe tapping, and gait is limited. By using a repetitive movement such as alternating toe tapping that is more closely indicative of gait may provide further insight into the relationship between bradykinesia and gait.

The use of external cueing has been a treatment strategy for both bradykinesia and gait (for review, Cassimatis et al., 2016). Thus, there has been substantial research on the effects of cueing, specifically auditory cueing, on unilateral repetitive finger tapping, unilateral repetitive toe tapping, and gait in persons with PD. Interestingly, previous research has suggested that cueing affects unilateral repetitive finger tapping, unilateral repetitive toe tapping, and gait similarly (Horin et al., 2021) adding support to the notion that impairments in these movements may be related. Both music and auditory cues at medium and fast tempi enable entrainment of unilateral finger tapping, unilateral toe tapping and stepping on a spot (Rose et al., 2019). However, spontaneous motor tempo was faster and more variable in persons with PD compared to healthy adults for both unilateral finger and toe tapping (Rose et al., 2020). These studies have manipulated the type of auditory cue and the rate of the cue, but there is limited evidence examining which side should be cued. For example, when cueing gait, the more affected side, the less affected side, or both sides can be cued. Moreover, it is unknown if movement performance is similar between repetitive movements and gait when the side cued is varied. Additional research teasing out these additional components is needed to further the knowledge regarding the relationship between bradykinesia, specifically repetitive toe tapping, and gait, as well as the use of auditory cueing for these movements in persons with PD.

The purpose of this study was (1) to compare the effects of auditory cueing on the more affected side (MAS), less affected side (LAS), and both sides during bilateral repetitive toe tapping and during gait, and (2) to determine if there was a relationship between bilateral repetitive toe tapping and gait for the cueing conditions. Given the paucity of research on which side should be cued during repetitive movements and gait, we hypothesize that there will be no difference between the cueing conditions. However, given the research supporting a relationship between bilateral repetitive finger movements and gait, we hypothesize that there will be a significant association between bilateral repetitive toe tapping performance and gait performance.

## 2. Methods

### 2.1. Participants

Twenty-seven participants (62% female, mean age ± standard deviation = 69 ± 8 years; 100% right handed; 100% white; mean education ± standard deviation = 15 ± 3 years; mean disease duration ± standard deviation = 7 ± 6 years; 45% with right side as more affected side) with PD were recruited for the study (Table 1). There were no exclusion criteria for this study, as the aim was to recruit a diverse population of persons with PD. All participants were tested on their optimal medication as prescribed by their treating physician. All participants gave their written informed consent prior to inclusion in the study, and the Institutional Review Board of Iowa State University approved the procedures.

**Table 1:** Participant demographics.

Participant	Gender	Age (years)	Disease duration (years)	More affected side
1	Female	77	6	Right
2	Male	84	11	Right
3	Female	49	5	Right
4	Female	79	15	Right
5	Female	72	11	Left
6	Female	68	7	Left
7	Male	71	1	Right
8	Male	65	6	Left
9	Male	74	11	Left
10	Female	62	2	Right
11	Female	83	2	Right
12	Female	67	6	Left
13	Female	82	2	Right
14	Female	65	13	Left
15	Female	75	3	Right
16	Male	74	7	Left
17	Male	68	2	Left
18	Female	62	6	Right
19	Female	59	7	Left
20	Female	48	16	Right
21	Female	76	10	Right
22	Male	65	1	Right
23	Female	82	23	Right
24	Male	67	1	Right
25	Female	78	1	Left
26	Female	62	1	Left
27	Female	56	15	Right

### 2.2. Data collection and analysis

**Figure 1:**
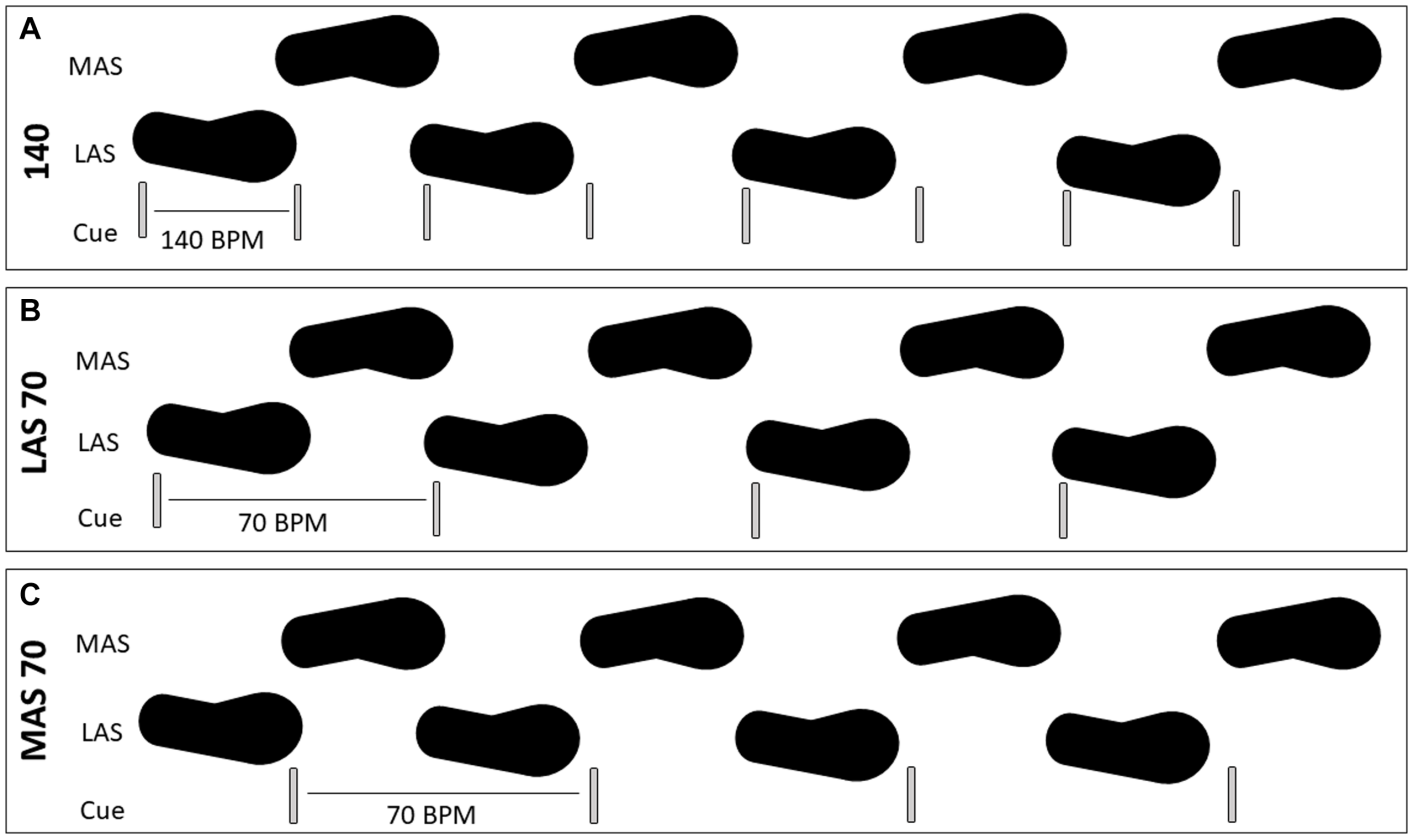
Schematic of the cueing paradigm used for both toe tapping and gait tasks. Cueing for both sides, with one beat per tap/step, at 140 beats per minute (BPM) is shown in **(A)**. Cueing for the less affected side (LAS), with one beat for the tap/step on the LAS, at 70 BPM is shown in **(B)**. Cueing for the more affected side (MAS), with one beat for the tap/step on the MAS, at 70 BPM is shown in **(C)**.

For the toe tapping tasks, participants completed alternating tapping for 10 s for each trial. Ten seconds was chosen to limit fatigue and was also limited by the data collection software parameters. Participants were instructed to keep their heel on the ground and tap only their toe in time with the given auditory cue. For gait, participants were instructed to step in time with the given auditory cue while walking across a 10-foot long GAITRite mat (CIR Systems, Inc.). Three cueing conditions were completed for both alternating toe tapping and gait. For the first condition, participants were provided an auditory cue at 70 beats per minute (BPM) and were asked to synchronize the MAS with the cue. For the second condition, participants were provided an auditory cue at 70 BPM and were asked to synchronize the LAS with the cue. For the third condition, participants were provided an auditory cue for both sides. The cue was paced at 140 BPM. These rates were chosen as previous literature has shown that impairments in repetitive movements emerge around 120 BPM and higher (Stegemöller et al., 2009, 2010, 2013, 2015, 2018, 2019). Four trials for each condition were collected to allow for collection of enough movements/stride for accurate analysis. See Figure 1 for schematic of the conditions. All trials for the toe tapping tasks were complete before the gait tasks. However, the cueing conditions were randomized for each task. Participants were allowed a practice trial for each condition.

For the toe tapping tasks, an electromagnetic sensor (Ascension) was placed on the top of the big toe on each foot and was recorded at 200 Hz. Peak flexion was marked for each movement to obtain peak amplitude. Inter movement interval was calculated as the time between each peak. The mean and standard deviation were calculated for each cueing condition. Coefficient of variability was then calculated (standard deviation/mean) for both amplitude and inter movement interval. For the gait tasks, participants walked across the GaitRite mat. Stance time, swing time, step length, and step width were obtained. The mean and standard deviation were calculated for each cueing condition.

### 2.3. Statistical analysis

For statistical analysis to test the first hypothesis, a one-way repeated measures analysis of variance was completed for each outcome measure to determine if there were differences between each cueing condition (MAS 70, LAS 70, 140) for both toe tapping and gait. *Post hoc* analyses were completed using paired t-tests with Bonferonni correction. To test the second hypothesis, two difference values were calculated to control for differences in cueing tempo (140 vs. 70); one between the 140 condition and the MAS 70 condition and one between the 140 condition and LAS 70 condition for each outcome measure. A Shapero-Wilk test revealed that the difference values were not normally distributed. Spearman correlations were then completed between the corresponding toe tapping tasks and gait tasks (140-MAS 70 toe tapping and 140-MAS 70 gait; 140-LAS 70 toe tapping and 140-LAS 70 gait).

## 3. Results

In general, for both toe tapping and gait, results revealed that performance was similar between the cueing conditions in which only one side was cued, regardless if it was the MAS or LAS. Differences emerged when comparing cueing on one side to cueing both sides.

**Figure 2:**
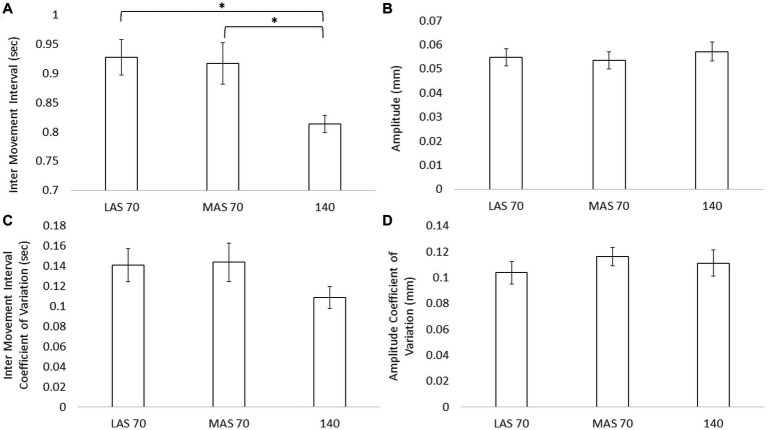
Mean and standard deviation for inter movement interval **(A)**, amplitude **(B)**, inter movement interval coefficient of variation **(C)**, and amplitude coefficient of variation **(D)** across the three cueing conditions, less affect side (LAS) cued at 70 beats per minute (BPM), more affected side (MAS) cues at 70 BPM, and both side cued at 140 BPM. Asterisks designate significant differences between conditions (*p* < 0.05).

Results for toe tapping are shown in Figure 2. Statistical analysis revealed a significant main effect for inter movement interval only [*F*(2) = 6.59, *p* = 0.003]. *Post hoc* analysis revealed differences between LAS70 and 140 cueing conditions [*t*(25) = 3.33, *p* = 0.003] and between MAS70 and 140 cueing conditions [*t*(25) = 2.69, *p* = 0.012]. There were no differences between the LAS70 and MAS70 cueing conditions [*t*(25) = 0.34, *p* = 0.74]. There were no main effects for all other outcome measures for toe tapping [Inter movement interval CV: *F*(2) = 1.82, *p* = 0.17; Amplitude: *F*(2) = 0.81, *p* = 0.45; Amplitude CV: *F*(2) = 0.48, *p* = 0.62].

**Figure 3:**
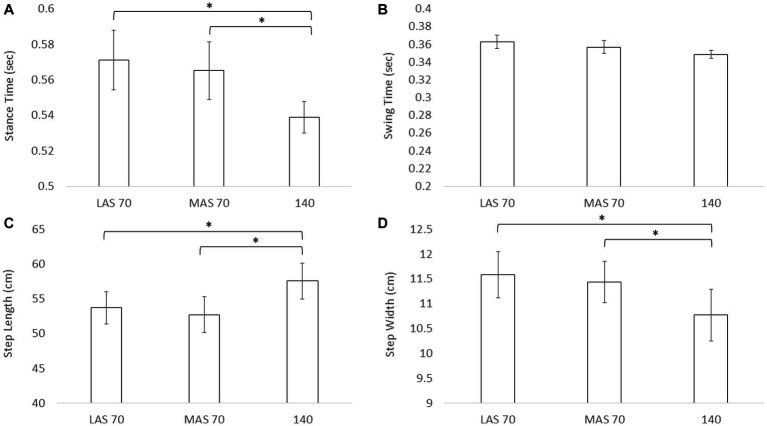
Mean and standard deviation for stance time **(A)**, swing time **(B)**, step length **(C)**, and step width **(D)** across the three cueing conditions, less affect side (LAS) cued at 70 beats per minute (BPM), more affected side (MAS) cues at 70 BPM, and both side cued at 140 BPM. Asterisks designate significant differences between conditions (*p* < 0.05).

Results for gait are shown in Figure 3. Statistical analysis revealed a significant main effect for stance time [*F*(2) = 5.43, *p* = 0.01], step length [*F*(2) = 6.49, *p* = 0.003], and step width [*F*(2) = 6.08, *p* = 0.004]. No main effect was revealed for swing time [*F*(2) = 3.04, *p* = 0.06]. *Post hoc* analysis for stance time, step length, and step width revealed the same pattern in which LAS70 differed from the 140 cueing condition, and MAS70 differed from the 140 cueing condition, but LAS70 and MAS70 cueing conditions did not differ. See Table 2 for statistical results.

**Table 2:** *Post hoc* analyses for gait.

	Stance time (*df* = 26)	Step length (*df* = 26)	Step width (*df* = 26)
LAS 70 vs. 140 (*t, p*)	−2.69, 0.01	−2.68, 0.01	−2.85, 0.009
MAS 70 vs. 140 (*t, p*)	−3.05, 0.005	−3.08, 0.005	−3.07, 0.005
LAS70 vs. MAS 70 (*t, p*)	0.57, 0.57	0.81, 0.42	0.61, 0.55

MAS, more affected side; LAS, less affected side; df, degrees of freedom. Shaded cells indicate significant differences.

Spearman correlations were completed between the difference scores for MAS70 and 140 and LAS70 and 140 for each toe tap and gait outcome measure and are shown in Table 3. For the MAS and 140 difference scores, no significant correlations were revealed between any of the toe tap and gait outcome measures. For the LAS and 140 difference scores, significant correlations between inter movement interval and stance time (*R* = 0.53, *p* = 0.005) and inter movement interval and step length (*R* = 0.45, *p* = 0.02) were revealed. There were no other significant correlations between the toe tap and gait outcome measures.

**Table 3:** Spearman correlations.

	Stance time	Swing time	Step length	Step width
MAS 70–140 (*N* = 26)				
Inter movement interval (*R, p*)	0.30, 0.13	0.30, 0.13	0.29, 0.15	0.05, 0.82
Inter movement interval CV (*R, p*)	−0.28,0.17	−0.24, 0.24	−0.11, 0.59	−0.14, 0.50
Amplitude (*R, p*)	−0.13, 0.52	−0.004, 0.98	−0.07, 0.75	0.22, 0.27
Amplitude CV (*R, p*)	−0.32, 0.11	−0.28, 0.16	−0.24, 0.25	0.13, 0.52
LAS 70–140 (*N* = 26)				
Inter movement interval (*R, p*)	0.53, 0.005	0.32, 0.11	0.45, 0.02	0.004, 0.98
Inter movement interval CV (*R, p*)	0.0, 0.99	−0.03, 0.89	0.13, 0.54	0.09, 0.65
Amplitude (*R, p*)	−0.01, 0.96	−0.15, 0.47	0.05, 0.82	0.14, 0.5
Amplitude CV (*R, p*)	−0.13, 0.53	0.07, 0.74	0.18, 0.38	−0.14, 0.49

MAS, more affected side; LAS, less affected side; CV, coefficient of variation. Shaded cells indicate significant correlations.

## 4. Discussion

The purpose of this study was two-fold; to compare cueing strategies for toe tapping and gait, and to determine if toe tapping and gait performance were correlated. We hypothesized that there would be no differences between cueing conditions. However, results revealed that cueing only one side resulted in longer (i.e., slower) inter movement intervals compared to cueing both sides during toe tapping. There was no effect of cueing on amplitude. For gait, a similar pattern emerged in which stance time was longer, step length was shorter, and step width was longer when cueing one side compared to cueing both sides. There was no effect on swing time. Moreover, these effects were the same whether cueing the MAS or LAS for both toe tapping and gait. These results suggest that cueing does matter, and that cueing both sides may be more beneficial that only cueing one side. Given the similarity in response to cueing between toe tapping and gait, this supports our hypothesis that toe tapping and gait performance would be related. However, results revealed that only step length and stance time (gait outcome measures) were correlated to inter movement interval (toe tap outcome measure) for the LAS70-140 condition. Thus, whether repetitive movements, such as toe tapping, maintain the same underlying impairment as gait, is only partially supported by these results.

### 4.1. Cueing

There is limited research examining which side to cue during both repetitive movements and gait. Studies using cues for upper extremity repetitive movement typically cue either just the dominant side (Horin et al., 2021), the more affected side only (Stegemöller et al., 2009, 2010, 2013, 2015, 2018, 2019), the participant preferred side (Rose et al., 2020) or did not state (Rose et al., 2019). Only one study has compared performance of cued repetitive finger movement between the more and less affected side, revealing no difference in movement performance between sides (Stegemöller et al., 2016). The results of this paper are in keeping with this in that there were no differences in performance of repetitive toe tapping whether the more or less affected side was cued. However, the repetitive task in this study was a bilateral task. When comparing to other studies on bilateral repetitive movements, those using cues for upper extremity bilateral repetitive movement and cues for gait typically cued both sides (Howe et al., 2003; Ford et al., 2010; Vercruysse et al., 2012a,b; Ghai et al., 2018). Interestingly, our results indicate that cueing both sides may be more beneficial for movement timing. When the cue was given for just one side, movement timing was slower than the given cue. When provided for both sides, movement timing was more in time with the cue. This same pattern was extended to gait. Step length was greater, stance time was shorter, and step width was smaller. Compared to controls, persons with PD have shorter step length, longer stance time, and wider step width (Mirelman et al., 2019), suggesting that cueing both sides may be most beneficial for movement performance. However, this study was a within subjects comparison. Further studies using a control group are needed to determine if the changes in toe tapping and gait are indeed improvements.

### 4.2. Similarity between gait and toe tapping

The breadth of literature comparing repetitive movements and gait in Parkinson’s disease has focused on upper extremity movements and FOG (Nieuwboer et al., 2009; Vercruysse et al., 2012a,b; Barbe et al., 2014). The present study did not measure FOG. However, a more recent study in persons with PD without FOG has revealed that amplitude of bilateral finger tapping and stride length were related, as well as the improvement in both after medication (Syeda et al., 2022). Though cues were not used in the study by Syeda et al. (2022), and they examined repetitive bilateral finger tapping, the results are similar to results revealed in this study. Differences in step length and stance time were related to inter movement interval differences in toe tapping. However, it is important to consider that associations were completed between the difference in movement performance from cueing one side to cueing both sides to control for cueing tempo. These results suggest that a similar mechanism may underlie the improvement in movement performance when cueing both sides for both toe tapping and gait, rather than a direct relationship between toe tapping and gait. Further study is needed to determine if these results hold when comparing toe tapping and gait across a wide and varied population of persons with PD and healthy controls. Moreover, while no episodes of FOG were observed during data collection for this study, results do suggest that there may also be a relationship between toe tapping and gait, providing initial evidence for further study of the relationship between toe tapping and FOG. Nonetheless, this study adds to the behavioral literature suggesting a shared mechanism between repetitive movements and gait.

### 4.3. Limitations

A limitation to this study is the lack of a healthy matched control group. This should be considered when interpreting the results. However, the differences revealed between cueing conditions and the associations between toe tapping and gait are new and novel adding to the literature. In addition, because there were no exclusion criteria, other possible co-morbidities, potential cognitive impairment, or potential hearing impairment may have contributed to the ability to complete the tasks. Participants were allowed practice trials and no data was excluded suggesting that participants understood the tasks and were able to complete the tasks effectively. Nonetheless, the lack of exclusion criteria should be considered when interpreting the results. Finally, given the small sample size and that participants were homozygous in terms of handedness, race, and education level, results may be less generalizable.

## 5. Conclusion

This study was the first to compare cueing of one side to cueing both sides, as well as exploring the relationship between toe tapping and gait in persons with PD. Results revealed that cueing both sides is better than only one side and that there is a relationship between toe tapping and gait performance when both sides are cued in persons with PD. This study adds to the literature exploring possible shared mechanisms between bradykinesia and gait in persons with PD.

## Data availability statement

The raw data supporting the conclusions of this article will be made available by the authors, without undue reservation.

## Ethics statement

The studies involving humans were approved by the Iowa State University Institutional Review Board. The studies were conducted in accordance with the local legislation and institutional requirements. The participants provided their written informed consent to participate in this study.

## Author contributions

ES: formal analysis, project administration, resources, supervision, and writing original draft. RB: data curation, formal analysis, writing review, and editing of draft. AW and MH: data curation, investigation, writing review, and editing of draft. All authors contributed to the article and approved the submitted version.

## Conflict of interest

The authors declare that the research was conducted in the absence of any commercial or financial relationships that could be construed as a potential conflict of interest.

## Publisher’s note

All claims expressed in this article are solely those of the authors and do not necessarily represent those of their affiliated organizations, or those of the publisher, the editors and the reviewers. Any product that may be evaluated in this article, or claim that may be made by its manufacturer, is not guaranteed or endorsed by the publisher.
